# Popliteal Aneurism Successfully Treated by the Flexion of the Limb, after a Failure of Treatment by Compression

**Published:** 1864-08

**Authors:** S. Curey

**Affiliations:** Deputy Inspector-General of Hospitals.


*Popliteal Aneurism successfully treated by the flexion of the limb, after failure of treatment by compression.* By S. Currey, M. D., C. B., Deputy Inspector-Gen. of Hospitals.

Compression was tried by the application of two of Sejourni's tourniquets, one over Scarpa's space, and the other lower down over the artery; two instruments being used with the view of mitigating the pain, by the alternate tightening and relaxation of the instruments.

The repeated application of the tourniquets produced the following effects: great emaciation of the thigh, increased hardness of the tumor, and œdema of the leg. On removing the pressure, the pulsation invariably returned with undiminished force.

The treatment by compression having failed, I decided upon trying flexion of the leg upon the thigh, combined with more generous fare. This was done by gradually flexing the leg, and after a few days bringing the heel in contact with the nates, and retaining it in that position by means of a web strap and buckle. This had the immediate effect of arresting the circulation in the tumor, to the great relief of the patient. He was allowed to move about on crutches, the foot being supported by a long string worn over the shoulders. On the ninth day the leg was extended to the semi-flexed, and on carefully examining the aneurism the pulsation was found to be feeble, and the bruit almost inaudible. On each occasion that the leg was extended for the purpose of ascertaining the progress of the case, the returning pulsation in the tumor, though feeble, could be distinctly felt. Though the collateral circulation soon became very distinct and strong, the treatment had only been attended with a tantalizing amount of success—just sufficient to encourage the hope that perseverance would eventually be rewarded with a perfect cure. At length, despairing of being able to effect complete consolidation of the tumor, it was deemed advisable to abandon the flexion process, and to have recourse to ligature of the artery. As a preparatory step to this operation, the strap was removed and the limb straightened as much as practicable, for a considerable degree of contraction of the knee had taken place from long continued flexion. On returning to the patient the following evening, the unlooked-for discovery was made that the pulsations of the tumor had entirely ceased, and careful examination with the stethescope failed to detect the faintest bruit. In respect to the further history of the case, from this time there was no return of pulsation, and the cure in every respect was complete.

